# GroEL1, a Heat Shock protein 60 of *Chlamydia pneumoniae*, Impairs Neovascularization by Decreasing Endothelial Progenitor Cell Function

**DOI:** 10.1371/journal.pone.0084731

**Published:** 2013-12-23

**Authors:** Yi-Wen Lin, Chun-Yao Huang, Yung-Hsiang Chen, Chun-Ming Shih, Nai-Wen Tsao, Cheng-Yen Lin, Nen-Chung Chang, Chien-Sung Tsai, Hsiao-Ya Tsai, Jui-Chi Tsai, Po-Hsun Huang, Chi-Yuan Li, Feng-Yen Lin

**Affiliations:** 1 Department of Internal Medicine, School of Medicine, College of Medicine, Taipei Medical University, Taipei, Taiwan; 2 Division of Cardiology, Department of Internal Medicine and Cardiovascular Research Center, Taipei Medical University Hospital, Taipei, Taiwan; 3 Graduate Institute of Integrated Medicine, China Medical University, Taichung, Taiwan; 4 Division of Cardiovascular Surgery, Taipei Medical University Hospital, Taipei, Taiwan; 5 Department of Computer Science and Information Management, Hung Kuang University, Taichung, Taiwan; 6 Division of Cardiovascular Surgery, National Defense Medical Center, Taipei, Taiwan; 7 Division of Cardiology, Taipei Veterans General Hospital, Taipei, Taiwan; 8 Graduate Institute of Clinical Medical Sciences, China Medical University, Taichung, Taiwan; 9 Department of Anesthesiology, China Medical University Hospital, Taichung, Taiwan; European Institute of Oncology, Italy

## Abstract

The number and function of endothelial progenitor cells (EPCs) are sensitive to hyperglycemia, hypertension, and smoking in humans, which are also associated with the development of atherosclerosis. GroEL1 from *Chlamydia pneumoniae* has been found in atherosclerotic lesions and is related to atherosclerotic pathogenesis. However, the actual effects of GroEL1 on EPC function are unclear. In this study, we investigate the EPC function in GroEL1-administered hind limb-ischemic C57BL/B6 and C57BL/10ScNJ (a toll-like receptor 4 (TLR4) mutation) mice and human EPCs. In mice, laser Doppler imaging, flow cytometry, and immunohistochemistry were used to evaluate the degree of neo-vasculogenesis, circulating level of EPCs, and expression of CD34, vWF, and endothelial nitric oxide synthase (eNOS) in vessels. Blood flow in the ischemic limb was significantly impaired in C57BL/B6 but not C57BL/10ScNJ mice treated with GroEL1. Circulating EPCs were also decreased after GroEL1 administration in C57BL/B6 mice. Additionally, GroEL1 inhibited the expression of CD34 and eNOS in C57BL/B6 ischemic muscle. *In vitro*, GroEL1 impaired the capacity of differentiation, mobilization, tube formation, and migration of EPCs. GroEL1 increased senescence, which was mediated by caspases, p38 MAPK, and ERK1/2 signaling in EPCs. Furthermore, GroEL1 decreased integrin and E-selectin expression and induced inflammatory responses in EPCs. In conclusion, these findings suggest that TLR4 and impaired NO-related mechanisms could contribute to the reduced number and functional activity of EPCs in the presence of GroEL1 from *C. pneumoniae*.

## Introduction

*Chlamydia pneumoniae* is thought to play key roles in atherogenesis [1]. In clinical observation, *C. pneumoniae* accumulates in early fatty streaks in association with increasing vascular intima-media thickness [2]. Higher levels of plasma intercellular adhesion molecule-1 (ICAM-1), vascular cell adhesion molecule-1 (VCAM-1), and E-selectin in community-dwelling subjects [3] and in coronary arterial disease patients [4] are associated with *C. pneumoniae* seropositivity. In animal studies, inoculation of animals with *C. pneumoniae* may induce low-density lipoprotein (LDL) oxidation within the neointima [5], increase the formation of atherosclerotic lesions in hypercholesterolemic conditions [6] and accelerate the formation of complex atherosclerosis [7]. Indeed, the *in vitro* studies had confirmed these results which were obtained from clinical observation and animal studies. These evidence indicated that *C. pneumoniae* may increase uptake of LDL in macrophages [8,9], induce matrix metalloproteinases (MMPs) and adhesion molecules expression through activation of the lectin-like oxidized LDL receptor (LOX)-1 in human vascular endothelial cells [10-12], which may potentially promote the development of atherosclerosis. 

Heat shock protein 60 of *C. pneumoniae* (GroEL) is also expressed on the surface of elementary bodies (EBs), which are required for both attachment to and phagocytosis by host cells [13]. GroEL1 can fall off of the EBs and act as a major adhesion protein, playing an important role in the pathogenesis of *C. pneumoniae*-related diseases [14], including respiratory tract diseases and vascular diseases [15]. During inflammatory process, GroEL1 may initiate the secretion of interleukin (IL)-6, IL-1β, IL-8, and tumor necrosis factor (TNF)-α in vascular cells, mononuclear cells and dendritic cells [16-18]. In addition to our previous evidence demonstrating that GroEL1 induces LOX-1 [12,19] and VCAM-1 [19,20] expression in endothelial cells and enhances atherogenesis in hypercholesterolemic rabbits, the detailed mechanisms by which GroEL1 contributes to the critical process of atherogenesis need to be elucidated. 

Recent evidence suggests that endothelial dysfunction and injury of the vascular wall are repaired by endothelial progenitor cells (EPCs) [21]. The bone marrow-derived CD133^+^/CD34^+^/KDR^+^ EPCs may migrate to sites of damaged endothelium followed by differentiation into endothelial cells [22], thereby improving blood flow [22,23]. Two types of EPCs, early and late (late-outgrowth ) EPCs, can be derived and identified from peripheral blood [24,25] and mediate different roles in neovasculogenesis. Early EPCs could be an indicator of atherogenesis [26-29], and late EPCs may further contribute to vascular repair and angiogenesis [29-31]. A reduced number and function of EPCs are also associated with the development of atherosclerosis [32]. In humans with hyperglycemia or hypertension and in smokers, the number and function of circulating EPCs are impaired [33]. Persistent or excessive systemic inflammation results in the production of pro-inflammatory factors, such as TNF-α [34], IL-1β [35], granulocyte macrophage-colony stimulating factor (GM-CSF), and stromal-derived factor-1 (SDF-1), which are implicated in the pathology of cardiovascular diseases [36] and modulate EPC mobilization, recruitment, and homing [21,32].

 Although *C. pneumoniae* GroEL1 may disturb the endothelium and vessels [37,38], there are no reports demonstrating the effects of GroEL1 on EPCs function.

Therefore, in this study, we used human EPCs *in vitro* and a mouse hind limb ischemia model to explore the effects of GroEL1 on EPC function and the underlying mechanism.

## Materials and Methods

### Animals and GroEL1 Administration

All male C57BL/6 mice were purchased from BioLASCO Taiwan Co., Ltd. The male C57BL/10ScNJ mice (with a spontaneous toll-like receptor 4 mutation; homozygous for the defective lipopolysaccharide response, deletion allele *Tlr4* ^lps-del^) were purchased from the Jackson Laboratory (JAX®, 003752, Bar Harbor, ME, USA). All animals were treated according to protocols approved by the Institutional Animal Care Committee of the Taipei Medical University (Taipei, Taiwan). The experimental procedures and animal care conformed to the “Guide for the Care and Use of Laboratory Animals” published by the U.S. National Institutes of Health (NIH Publication No. 85-23, revised 1996). All mice were kept in microisolator cages on a 12-h day/night cycle and fed a commercial mouse chow diet (Scientific Diet Services, Essex, UK) with water *ad libitum*. Thirty C57BL/B6 and 18 C57BL/10ScNJ mice were used. C57BL/B6 mice were included in groups 1-5, and C57BL/10ScNJ mice were included in groups 6-8. Group 1 (C57BL/B6 mice; naïve) was the naïve control group; group 2 (C57BL/B6 mice; IS) received a hind limb ischemia operation at the beginning of week 1 of the experiment; group 3 (C57BL/B6 mice; IS+GroEL1 1 μg/kg BW) received a hind limb ischemia operation at the begin of week 1 and tail vein injection of 1 μg/kg body weight (BW) of GroEL1 once a week throughout the experiment (8 weeks); group 4 (C57BL/B6 mice; IS+GroEL1 2 μg/kg BW) received a hind limb ischemia operation at the beginning of week 1 and tail vein injection of 2 μg/kg BW of GroEL1; group 5 (C57BL/B6 mice; IS+GroEL1 4 μg/kg BW) received a hind limb ischemia operation at the beginning of week 1 and tail vein injection of 4 μg/kg BW of GroEL1; group 6 (C57BL/10ScNJ mice; naïve) was the naïve control group; group 7 (C57BL/10ScNJ mice; IS) received a hind limb ischemia operation at the beginning of week 1 of the experiment; group 8 (C57BL/10ScNJ mice; IS+GroEL1 4 μg/kg BW) received a hind limb ischemia operation at the beginning of week 1 and tail vein injection of 4 μg/kg body weight (BW) of GroEL1.

### Mouse Ischemic Hind Limb Model

At the beginning of the experiments, unilateral hind limb ischemia was induced in six-week-old mice by ligating and excising the right femoral artery as previously described [39]. Briefly, the animals were anesthetized by intraperitoneal injection of Xylocaine (2 mg/kg BW) plus Zoletil (5 mg/kg BW; containing a dissociative anesthetic, Tiletamine/Zolazepam, at a ratio of 1:1). The proximal and distal portions of the femoral artery were ligated by silk thread, and we cut the blood vessel approximately 0.2 cm. Hind limb blood perfusion was measured with a laser Doppler perfusion imager system (Moor Instruments Limited, Devon, UK) before and after the surgery and was then followed up once every two weeks. The animals were sacrificed by cervical dislocation without sedation at the end of the eight experimental weeks. To avoid the influence of ambient light and temperature, the results are expressed as the ratio of perfusion in the right (ischemic) versus left (non-ischemic) limb.

### Flow Cytometry

To investigate the mobilization of EPCs, a fluorescence-activated cell sorting (FACS) Caliber flow cytometer (Becton Dickinson, San Jose, CA, USA) was used. Total leukocyte was isolated from a volume of 100-200 µL peripheral blood by incubating with 200-400 µL RBC lysis buffer, and washing twice in phosphate-buffered saline (PBS). Then total leukocyte was incubated with fluorescein isothiocyanate (FITC)-conjugated anti-mouse CD34 (eBioscience, San Diego, CA, USA), allophycocyanin (APC)-conjugated anti-mouse Flk-1 (VEGFR-2, eBioscience, San Diego, CA, USA), and phycoerythrin (PE)-conjugated anti-mouse Sca-1 antibodies (eBioscience, San Diego, CA, USA). Isotype-identical antibodies served as controls (Becton Dickinson, Franklin Lakes, NJ, USA). Each analysis included 150,000~300,000 total leukocyte. Circulating EPCs were considered to be from the monocytes population and were gated with triple positivity for CD34, Sca-1, and Flk-1.

### Immunohistochemistry

The whole ischemic limbs were harvested; the adhering tissues and femora were carefully removed, and samples were immersion-fixed with 4% buffered paraformaldehyde, performed on serial 5-μm-thick paraffin-embedded sections. Immunohistochemical staining was performed on mouse ischemic thigh muscle (sartorius muscle, gracilis muscle, adductor muscle and semimembranosus muscle were included) using goat anti-Von Willebrand factor (vWF; Santa Cruz Biotechnologies, Santa Cruz, CA, USA), rabbit anti-CD34 (Millipore, MA, USA), and goat anti-endothelial NO synthase antibodies (eNOS; Cell Signaling, CA, USA), followed by counterstaining with Hoechst. The stained slides were observed using fluorescence microscopy. Three cross-sections were analyzed for each animal, 10 different fields from each tissue preparation were randomly selected, and visible capillaries were counted. Capillary density is expressed as the capillary/myofiber ratio.

### Reagents and Antibodies

Carbobenzoxy-valyl-alanyl-aspartyl-[O-methyl]-fluoromethylketone (Z-VAD-FMK, a cell-permeant pan-caspase inhibitor), Ac-Leu-Glu-His-Asp-chloromethylketone (Ac-LEHD-CMK, a specific caspase-9 inhibitor), and Ac-Ile-Glu-Thr-Asp-aldehyde (Ac-IETD-CHO, a specific caspase-8 inhibitor) were purchased from BD Biosciences (San Jose, CA, USA). SB203580 (a p38 MAPK inhibitor), PD98059 (a ERK1/2 inhibitor), PD98059 (a JNK/SAPK inhibitor), rabbit anti-p38 MAPK antibody, rabbit anti-phospho-p38 MAPK antibody, rabbit anti-JNK/SAPK antibody, rabbit anti-phospho-JNK/SAPK antibody, rabbit anti-ERK1/2 antibody, mouse anti-phospho-ERK1/2 antibody, mouse anti-human phospho-endothelial nitric oxide synthase (eNOS) antibody, anti-human eNOS antibody, mouse anti-caspase-8 antibody and mouse anti-caspase-3 antibody were purchased from Cell Signaling Technology Co. (Beverly, MA, USA). The mouse anti-human VCAM-1 and mouse anti-human ICAM-1 antibodies were purchased from R&D Systems (Minneapolis, MN, USA). The rabbit anti-human caspase-9 antibody was purchased from EPITOMIC Co. (Burlingame, CA, USA). 

### Manufacture and Purification of *C. Pneumoniae* Recombinant GroEL1 Protein

*C. pneumonia* (TWAR TW-183) was cultured on HeLa 229 cells. The construction of GroEL1 expression vectors, purification of recombinant GroEL1 protein, and measurement of GroEL1 protein cytotoxicity and activity were described in our previous report [19].

### EPC Isolation and HCAEC Cultivation

Total mononuclear cells (MNCs) were isolated from 40 ml of peripheral blood from healthy young male volunteers by density-gradient centrifugation with Histopaq-1077 (density 1.077 g/mL; Sigma). The Institutional Review Board (Taipei Medical University-Joint Institutional Review Board) approved this study, and all volunteers gave written informed consent prior to all procedures. MNCs (1x10^7^ cells) were plated in 2 ml of endothelial growth medium (EGM-2 MV; Cambrex, Charles, IA, USA) with supplements (hydrocortisone, R3-insulin-like growth factor 1, human vascular endothelial growth factor, human fibroblast growth factor, gentamicin, amphotericin B, vitamin C, and 20% fetal bovine serum) on fibronectin-coated six-well plates at 37°C in a 5% CO_2_ incubator. The cultures were observed daily, and after 4 days of culture, the media were changed and nonadherent cells were removed; attached early EPCs were elongated, with a spindle shape. Thereafter, media were replaced every 3 days, and each colony/cluster was observed. A certain number of early EPCs continued to grow into colonies of late EPCs, which emerged 2-4 weeks after the start of MNC culture. The characterization of EPCs was described previously [31]. HCAECs were purchased from Cascade Biologics, Inc. (Portland, OR, USA). HCAECs were cultured in M200 medium (Cascade Biologics, Inc., Portland, OR, USA), and passages were performed according to the manufacturer’s instructions. 

### Early EPC Formation Assay

Isolated MNCs were resuspended in EndoCult Liquid Medium Kit (StemCell Technologies, Vancouver, Canada), and a total of 5x10^6^ MNCs were preplated in fibronectin-coated 6-well plates in duplicate. After 2 days, the suspension cells were collected and resuspended in EndoCult Liquid Medium Kit, and 1x10^6^ cells were replaced onto a fibronectin-coated 24-well plate. Cultured cells were then treated with GroEL1 for 4 days. On day four of the assay, the attached early EPCs were characterized as adherent cells that were dually positive for lactin staining and DiI-acLDL uptake. The early EPCs were counted manually in a minimum of three wells in high-power microscopic fields. All cells were incubated on the day of isolation from whole blood with or without GroEL1.

### Late EPC Tube Formation Assay

The tube formation assay was performed on EPCs to assess angiogenic capacity, which is involved in new vessel formation [40]. The *in vitro* tube formation assay was performed using the Angiogenesis Assay Kit (Chemicon, CA, USA) according to the manufacturer’s protocol. In brief, ECMatrix gel solution was thawed at 4°C overnight, mixed with ECMatrix diluent buffer, and placed in a 96-well plate at 37°C for 1 hour to allow the matrix solution to solidify. EPCs were treated with GroEL1 for 24 hours and then harvested. A total of 10^4^ cells were placed on the matrix solution with GroEL1, and the samples were incubated at 37°C for 12 hours. Tubule formation was inspected under an inverted light microscope. Four representative fields were taken, and the average of the total area of complete tubes formed by the cells was compared using Image-Pro Plus computer software.

### Late EPC Migration Assay

The migratory function of late EPCs, which is essential for vasculogenesis, was evaluated by a modified Boyden chamber (Transwell, Coster) assay [40]. Late EPCs were incubated with GroEL1 for 24 h. The 4x10^4^ treated EPCs were placed in the upper chamber of 24-well Transwell plates with a polycarbonate membrane (8 μm pores) with EGM-2 MV medium; VEGF (50 ng/mL) in EGM-2 MV medium was placed in the lower chamber. After incubation for 24 h, the membrane was washed briefly with PBS and fixed with 4% paraformaldehyde. The upper side of the membrane was wiped gently with a cotton ball. The membrane was then stained using hematoxylin solution and removed. The magnitude of migration of late EPCs was evaluated by counting the migrated cells in six random high-power (100x) microscope fields.

### Late EPC Proliferation Assay

The effect of GroEL1 on cell proliferation was analyzed by the 3-(4,5-dimethylthiazol-2-yl) -2,5-diphenyl tetrazolium bromide (MTT) assay. Late EPCs (2x10^4^ cells) were grown in 96-well plates and incubated with GroEL1 for 24 h. Subsequently, MTT (0.5 μg/mL) was applied to cells for 4 h. The cells were lysed with dimethyl sulfoxide (DMSO), and the absorbance was read at 530 nm by using a DIAS Microplate Reader (Dynex Technologies, VA, USA).

### Late EPC Adhesion Assay

Before the assay was performed, the 24-well plates were distributed with human coronary artery endothelial cells (HCAECs; 5x10^6^ cells were allowed to reach confluence) or coated with fibronectin and collagen (incubated with 10 μg/mL of fibronectin and collagen for 1 hour). Late EPCs were incubated with GroEL1 for 24 hours, followed by 5 μg/mL 20,70-bis(2-carboxyethyl)-5(6)-carboxy-fluorescein acetoxymethyl ester (BCECF/AM; Invitrogen, CA, USA) for 1 hour at 37°C in serum-free EGM-2 MV medium. Then, 10^6^ labeled late EPCs were added to each prepared well, and incubation continued for 1 h. Non-adherent cells were removed, and the cell extracts were prepared with DMSO. The fluorescence was quantified by fluorimetry (Wallac Victor^2^, Finland).

### Late EPC Senescence Assay

Senescence was characterized as a limited capacity of normal cells to replicate, meaning an arrested state in which the cell remained viable. Cellular aging was determined with a Senescent Cells Staining Kit (Sigma-Aldrich, CA, USA). Briefly, after treatment with GroEL1, EPCs were fixed in 2% formaldehyde and 0.2% glutaraldehyde and then incubated for 12 hours at 37°C in the presence of fresh X-gal staining solution. After staining, the blue-stained cells and the total number of cells were counted, and the percentage of β-galactosidase-positive cells was calculated.

### Western Blot Analysis

Cells were lysed with lysis buffer. The protein concentration in the supernatants was measured using a protein determination kit (Bio-Rad, CA, USA). The supernatants were subjected to 8% SDS-PAGE and transferred for 1 hour at room temperature to polyvinylidene difluoride membranes. The membranes were treated for 1 hour at room temperature with PBS containing 0.05% Tween-20 and 2% skim milk and incubated separately for 1 hour at room temperature with the primary antibodies. The membranes were incubated with horseradish peroxidase-conjugated IgG. Immunodetection was performed using a chemiluminescence reagent and exposure to Biomax MR Film (Kodak, NY, USA).

### Traditional Polymerase Chain Reaction and Quantitative Real-time Polymerase Chain Reactions

Total RNA was isolated using a TRIzol reagent kit (Invitrogen, CA, USA) according to the manufacturer’s instructions. The cDNA was synthesized from total RNA using Superscript^®^ II reverse transcriptase. Traditional PCR was performed using Promega PCR reagents. Glyceraldehyde 3-phosphate dehydrogenase (GAPDH) was amplified in the same samples to verify RNA abundance. The PCR mixture was amplified in a DNA thermal cycler (Biometra T3, Berlin, Germany) with 35 cycles for GAPDH and integrins. Quantitative real-time PCR was performed using a FastStart DNA Master SYBR Green I kit and a LightCycler (Roche, CA, USA). The levels of integrin α1, -α2, -β1, -β3, and E-selectin mRNAs were determined in arbitrary units by comparison with an external DNA standard. All specific primers were synthesized by Medclub Scientific Co., LTD. (Taoyuan, Taiwan). PCR primers used for amplification of integrins, E-selectin, and GAPDH were showed in the Table 1. 

**Table 1:** PCR primers were used for polymerase chain reactions.

Gene	forward/reverse
integrin α1	5’-tgc cat tat ggg tca tcc tgc tg-3’/5’-cac ata ttt gag gca aac ctg agg-3’
integrin α2	5’-cat caa cgt tcc aga cag tac agc-3’/5’-gct aac agc aaa agg att cca gc-3’
integrin β1	5’-ctg gtg tgg ttg ctg gaa ttg ttc-3’/5’-cct cat act tcg gat tga cca cag-3’
integrin β3	5’-cct gct cat ctg gaa act cct ca-3’/5’-cgg tac gtg ata ttg gtg aag gta g-3’
E-selectin	5’-ttg gta gct gga ctt tct gct gc-3’/5’-gta aga agg ctt ttg gta gct tcc-3’
GAPDH	5’-tgc ccc ctc tgc tga tgc c-3’/5’-cct ccg acg cct gct tca cca c-3’

GAPDH, glyceraldehyde 3-phosphate dehydrogenase

### Statistical Analyses

Values are expressed as the mean ± SEM. Statistical evaluation was performed using Student’s t test and one- or two-way ANOVA followed by Dunnett’s test. A probability value of p<0.05 was considered significant.

## Results

### GroEL1 Impairs Recovery of Capillary Density in C57BL/B6 But Not C57BL/10ScNJ Mice

**Figure 1:**
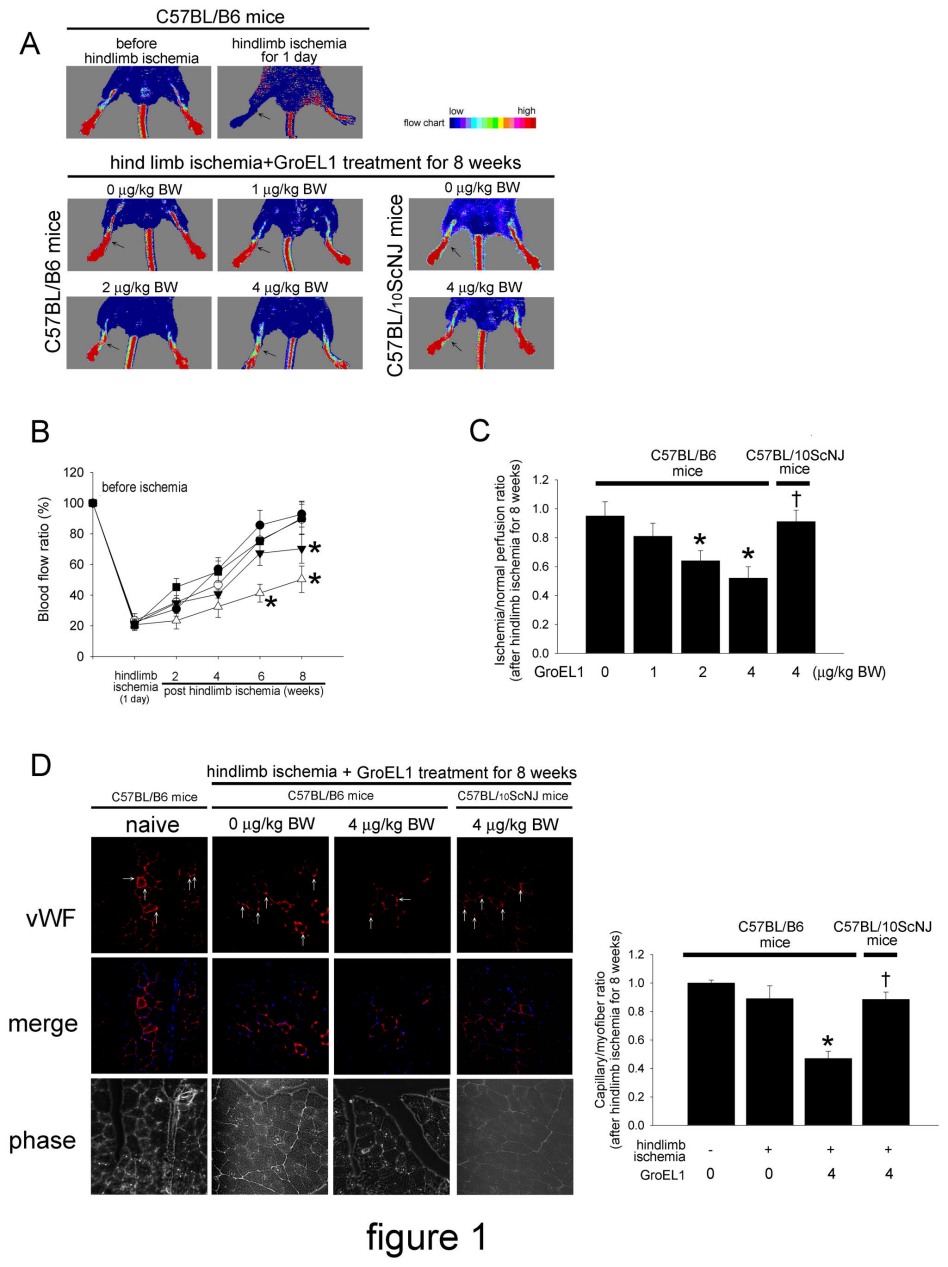
Effect of GroEL1 on blood flow recovery after hind limb ischemia in C57BL/B6 and C57BL/10ScNJ **mice**. (A) Upper, representative results of laser Doppler measurements before operation (control) and 1 day after hind limb ischemia surgery in C57BL/B6 mice. Lower, representative results of laser Doppler measurements 8 weeks after hind limb ischemia surgery in C57BL/B6 and C57BL/10ScNJ mice treated with 0-4 μg/kg BW of GroEL1. Color scale illustrates blood flow variations from minimal (dark blue) to maximal (red). Arrows indicate the ischemic (right) limb after hind limb ischemia surgery. (B) Doppler perfusion ratios (ischemic/non-ischemic hind limb) over time in the different groups. In C57BL/B6 mice, administration of 4 μg/kg BW (△) of GroEL1 or 2 μg/kg BW (▼) of GroEL1 impaired beneficial blood flow recovery compared with the non-administered group (●) 6 or 8 weeks after hind limb ischemia surgery. There was no significant difference in blood flow in the limb in the 1 μg/kg BW GroEL1-treated C57BL/B6 mice (○) or the 4 μg/kg BW GroEL1-treated C57BL/10ScNJ mice (█) compared with the non-administered C57BL/B6 mice (●). The results are expressed as the mean ± SEM (*p < 0.05 compared with non-GroEL1-treated C57BL/B6 mice at the same time point after ischemic surgery). (C) Eight weeks after ischemic surgery, the ischemia/normal perfusion ratio in the GroEL1-treated C57BL/B6 mice, but not GroEL1-treated C57BL/10ScNJ mice, was lower than that in the non-GroEL1-treated C57BL/B6 mice. The results are expressed as the mean ± SEM (n=6, *p < 0.05 compared with non-GroEL1-treated C57BL/B6 mice; †*p* < 0.05 compared with 4 μg/kg BW of GroEL1-treated C57BL/B6 mice). (D) Representative results of immunohistochemistry before operation (naïve) in C57BL/B6 mice. Mice were sacrificed 8 weeks after surgery, and capillaries (white arrow) in the ischemic muscles were visualized by anti-vWF immunostaining (original magnification x400). Hoechst dye (blue) was used to counterstain the nucleus. The graph shows the quantification of capillary density in hind limb-ischemic and GroEL1-administered C57BL/B6 and C57BL/10ScNJ mice. The results are expressed as the mean ± SEM (n=6, *p < 0.05 compared with hind limb ischemia+ non-GroEL1-treated C57BL/B6 mice; †*p* < 0.05 compared with hind limb ischemia+ 4 μg/kg BW of GroEL1-treated C57BL/B6 mice).

To evaluate the angiogenic effect of GroEL1, we induced tissue ischemia by performing a unilateral hind limb ischemia surgery on wild-type male C57BL/B6 and C57BL/10ScNJ mice (n = 6 for each group). Ligation and excision of the femoral artery effectively blocked blood flow in C57CL/B6 mice (Figure 1A). There was significant improvement of blood flow in mice treated only with hind limb ischemia (hind limb ischemia+GroEL1 0 μg/kg BW) 8 weeks post-ischemic surgery. The hind limb ischemic mice with 2 or 4 μg/kg BW of GroEL1 administration had delayed blood flow recovery after ischemia surgery compared with non-GroEL1-treated mice, as determined by laser Doppler imaging. Blood flow in ischemic limbs was significantly impaired at 8 weeks in the 2 μg/kg BW GroEL1-treated mice (75.24±8.90 % of non-ischemic hind limb at 8 weeks) and at 6 and 8 weeks in the 4 μg/kg BW GroEL1-treated mice (41.30±5.81 % of non-ischemic hind limb at 6 weeks and 50.32±8.62 % of non-ischemic hind limb at 8 weeks) compared with the non-ischemic hind limb (Figure 1B). In contrast, the hind limb-ischemic C57BL/10ScNJ mice with 4 μg/kg BW of GroEL1 administration did not have delayed blood flow recovery after ischemia surgery compared with non-GroEL1-treated C57BL/10ScNJ mice. Eight weeks following hind limb ischemic surgery, the ischemia/normal perfusion ratios were lower in the 2 and 4 μg/kg BW GroEL1-treated C57BL/B6 micecompared to the non-GroEL1-treated C57BL/B6 mice (non-GroEL1: 0.95±0.07, 2 μg/kg BW GroEL1: 0.64±0.07, and 4 μg/kg BW GroEL1: 0.52±0.08)(Figure 1C). However, the ischemia/normal perfusion ratio was not different between non-GroEL1-treated C57BL/B6 mice and 4 μg/kg BW GroEL1-treated C57BL/10ScNJ mice 8 weeks after hind limb ischemia surgery. Figure 1D shows the results of immunohistochemistry before operation (C57BL/B6: naïve) and 8 weeks after hind limb ischemia surgery (C57BL/B6 mice: hind limb ischemia+GroEL1 0 μg/kg BW). Consistent with the measurements obtained by laser Doppler imaging, anti-vWF immunostaining revealed that GroEL1 administration for 8 weeks at 4 μg/kg BW significantly decreased the number of detectable capillaries in the ischemic muscle of C57BL/B6 mice (Figure 1D) compared to untreated ischemic C57BL/B6 mice. In contrast, GroEL1 administration at 4 μg/kg BW did not significantly decrease the number of detectable capillaries in the ischemic muscle of C57BL/10ScNJ mice. 

### GroEL1 Protein Decreases Circulating EPCs and eNOS Expression As Well As New Small Vessel Formation at Sites of Ischemia in C57BL/B6 Mice

**Figure 2:**
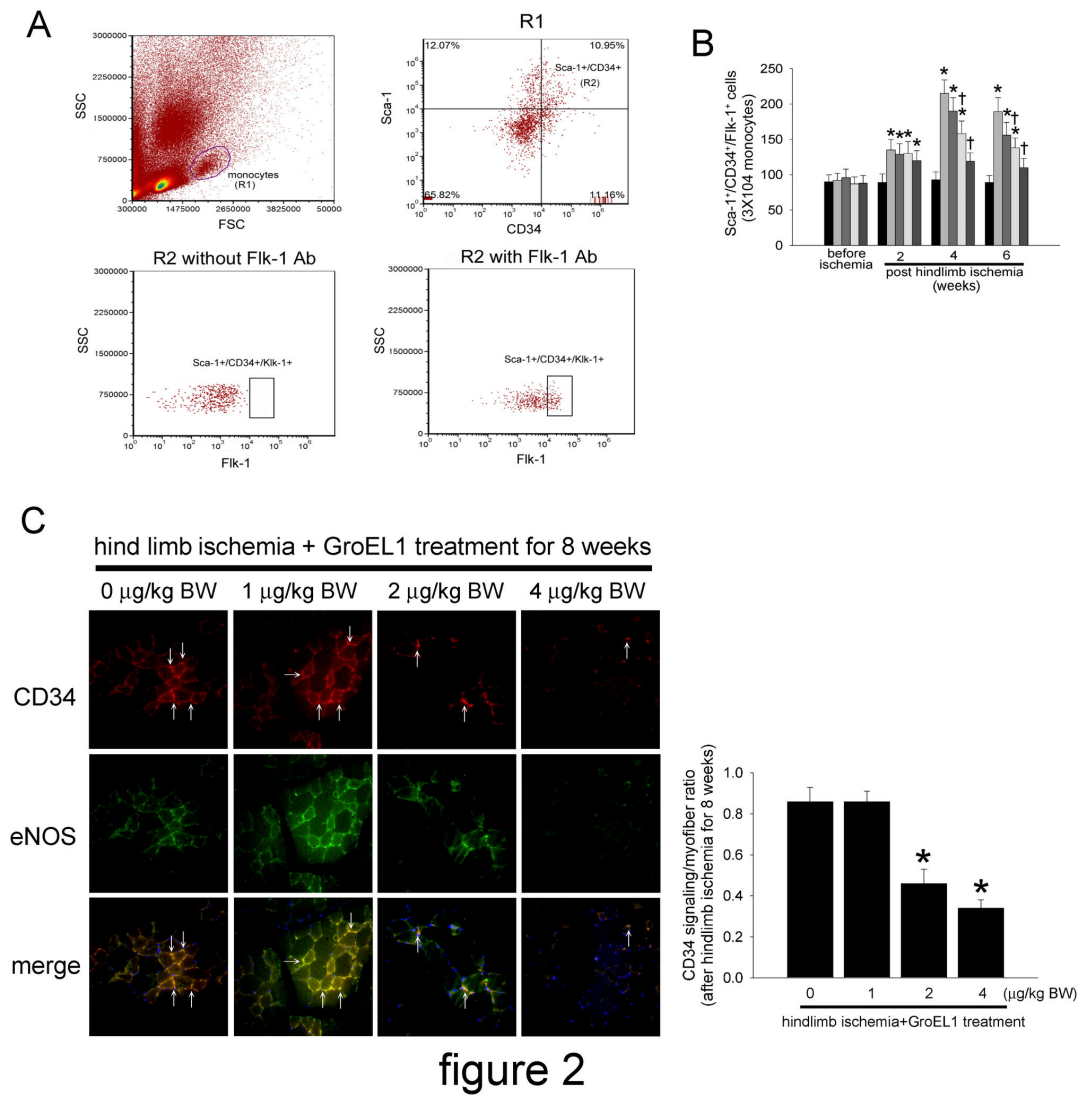
Effects of GroEL1 on EPC mobilization and eNOS expression at ischemic sites in hind limb-ischemic mice. (A) EPC was determined using flow cytometry. Forware SCatter (FSC) and Side SCatter (SSC) were used to gate the monocytes (R1). Hematopoietic stem cells were defined as Sca-1^+^/CD34^+^ cells (R2). EPCs were defined as Sca-1^+^/CD34^+^/Flk-1^+^ cells (R3). (B) The Sca-1^+^/CD34^+^/Flk-1^+^ cells in control (bar 1), non-GroEL1 (bar 2), 1 μg/kg BW GroEL1 (bar 3), 2 μg/kg BW GroEL1 (bar 4), and 4 μg/kg BW GroEL1 (bar 5)-administered mice 2 to 6 weeks after surgery. The results are expressed as the mean ± SEM (n=6, *p < 0.05 compared with naive C57BL/B6 mice at the same time point; †*p* < 0.05 compared with hind limb ischemia+non-GroEL1-treated C57BL/B6 mice at the same time point). (C) Immunostaining of ischemic hind limb muscle with anti-CD34 antibody conjugated to Alexa Fluor 633 (red) and anti-eNOS antibody conjugated to Alexa Fluor 488 (green) in C57BL/B6 mice treated with 1-4 μg/kg BW of GroEL1. The CD34-positive homed hematopoietic stem precursor cells were indicated with white arrows. By immunofluorescence staining, 2 μg/kg BW GroEL1 moderately decreased and 4 μg/kg BW GroEL1 severely decreased the number of eNOS/CD34-double-positive cells (arrow) in ischemic muscle compared with the non-GroEL1-treated group. Hoechst dye was used to counterstain the nucleus. The ischemic hind limb tissue was evaluated by fluorescence microscopy at a magnification of 400x. The bar graph shows the CD34 positivity/myofiber ratio. The results are expressed as the mean ± SEM (*p < 0.05 compared with hind limb ischemia+ non-GroEL1-treated C57BL/B6 mice).

To investigate the effects of GroEL1 stimulation on EPC mobilization in response to tissue ischemia, level of Sca-1^+^/CD34^+^/Flk-1^+^ cells in peripheral blood was determined using flow cytometry in C57BL/B6 mice. The basal number of EPCs did not differ significantly between individual groups. After surgery for 2 weeks, hind limb ischemia-administered C57BL/B6 mice had more circulating EPCs than the control (non-surgery) group; additionally, GroEL1 stimulation did not affect the circulating EPC level in hind limb-ischemic mice. However, treatment with GroEL1 decreased mobilization of EPCs in hind limb-ischemic C57BL/B6 mice 4 and 6 weeks after operation (Figure 2B). As seen in the diagram in Figure 2B, EPC mobilization was enhanced by tissue ischemia in control mice (baseline vs. 2 weeks after surgery: 89.5±12.3 cells vs. 135.7±15.5 cells per 3x10^4^ monocytes). At 4 and 6 weeks after hind limb ischemia surgery, C57BL/B6 mice from the GroEL1-treated groups exhibited a decreased capacity to mobilize EPCs into peripheral blood (1 μg/kg BW GroEL1: 190.4±19.4 cells at 4 weeks and 156.9±18.6 cells at 6 weeks; 2 μg/kg BW GroEL1: 158.9±21.4 cells at 4 weeks and 138.6±20.3 cells at 6 weeks; 4 μg/kg BW GroEL1: 110.7±18.4 cells at 4 weeks and 109.4±14.3 cells at 6 weeks) than the non-GroEL1-treated group (215.6±19.6 cells at 4 weeks and 189.3±21.6 cells at 6 weeks). 

As NO plays an important role in modulating EPC function, which is critical for blood vessel repair and angiogenesis [41], and CD34 is considered a critical marker of endothelial cells of small blood vessels, we next measured eNOS and CD34 in histological sections harvested from the ischemic hind limbs of C57CL/B6 mice. Consistent with our flow cytometry results, anti-CD34 immunostaining revealed that administration of 2 or 4 μg/kg BW of GroEL1 for 8 weeks significantly decreased the number of detectable capillaries in the ischemic thigh muscle of treated compared to untreated ischemic C57CL/B6 mice. Similar to CD34 expression, eNOS expression was visible in hind limb-ischemic C57CL/B6 mice. Administration of GroEL1 (2 or 4 μg/kg BW administration) actually decreased eNOS expression in hind limb-ischemic C57CL/B6 mice compared to non-GroEL1-treated hind limb-ischemic C57CL/B6 mice (Figure 2C). 

### GroEL1 Impairs the Differentiation and Mobilization Capacity of EPCs

**Figure 3:**
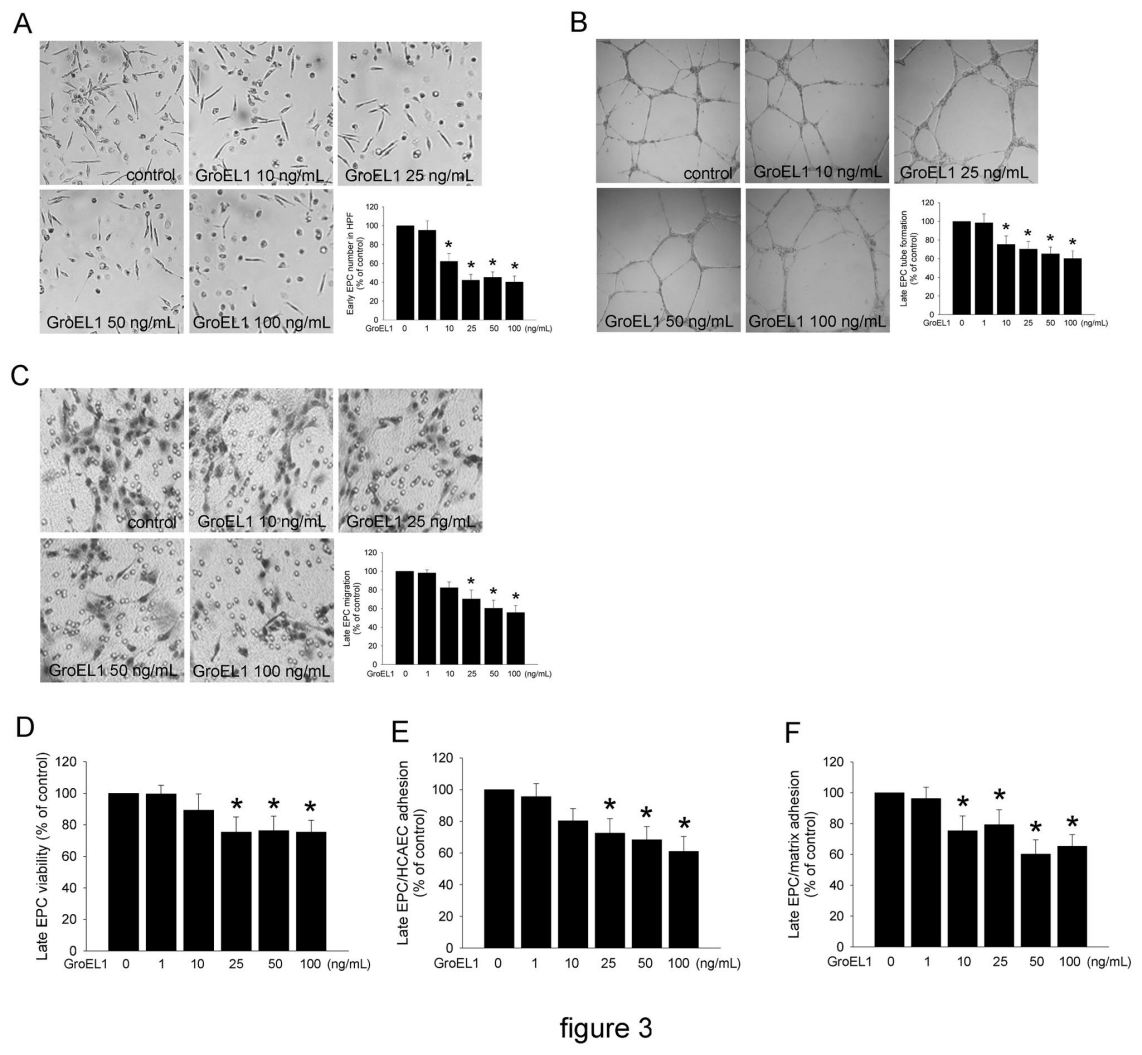
GroEL1 decreases EPC number and function. (A) The collected-human MNCs in growth medium were incubated with different concentrations of GroEL1 (1-100 ng/mL) for 4 days. Early EPCs are indicated with arrows. The numbers of EPCs were counted under microscope in a high-power field (HPF). (B) ECMatrix gel was used to assay the *in* *vitro* angiogenesis capacity of late EPCs. Representative photos of *in* *vitro* angiogenesis are shown. The mean total area of complete tubes formed by each GroEL1-treated group was calculated and compared with the non-GroEL1-treated group. (C) A modified Boyden chamber assay was used with VEGF as chemoattractive factor to evaluate late EPC migratory function. Representative photos are shown; the migrated cells were stained with hematoxylin and counted under microscope. (D) Late EPCs were treated with GroEL1 (1-100 ng/mL) for 24 hours. MTT assay was also performed to evaluate late EPC proliferation activity. (E and F) Late EPCs were preincubated for 24 hours with or without GroEL1, then labeled with BCECF/AM and attached to fibronectin/collagen-coated plates or HCAEC-cultured dishes for 1 hour. The attached EPCs were lysed using DMSO, and the fluorescence was quantified by fluorimetry. All data are expressed as the mean ± SEM (*p < 0.05 compared with untreated group).

Even though the characteristics of early EPCs are distinct from late EPCs, they also promote the differentiation and function of late EPCs [42,43]. Therefore, we tested the formation of early EPCs from GroEL1-conditioned mononuclear cells. On day 4 of assay, incubation of cells with 10, 25, 50, and 100 ng/mL GroEL1 significantly decreased the number of differentiated/adherent early EPCs (62.3±8.3%, 45.3±5.7%, 40.3±6.3%, and 42.1±6.3% of control, respectively) compared with the untreated group (Figure 3A). The capillary network formation and migratory function of EPCs are believed to be important during neovascularization [29,44]. An *in vitro* angiogenesis and migration assay was performed with late EPCs to investigate the effects of GroEL1 on EPCs. After 4 days of culturing, the functional capacity for tube formation of late EPCs on ECMatrix gel was significantly reduced in the 10, 25, 50, and 100 ng/mL GroEL1-treated groups (75.3±9.2%, 70.2±8.3%, 65.3±7.2%, and 60.2±8.3% of control, respectively) compared with the control group (Figure 3B). Similarly, the migratory ability was decreased in 25, 50, and 100 ng/mL GroEL1-stimulated late EPCs (70.2±9.6%, 60.3±8.5%, and 55.7±7.4% of control, respectively) (Figure 3C). To measure cell viability, the MTT assay was used to detect the number of viable and proliferating cells. Figure 3D shows that GroEL1 inhibited EPC proliferation activity, which became apparent at 25, 50 and 100 ng/mL (75.3±9.6%, 76.3±9.2%, and 75.3±7.6% of control, respectively). Adhesion to extracellular matrix and endothelial cells is now considered an essential step of the homing of EPCs to ischemic tissues [23,45]. Thus, we examined the effects of GroEL1 on EPC adhesion to HCAECs and to the fibronectin/collagen matrix. Stimulation of EPCs with GroEL1 significantly increased the adhesion of EPCs to HCAECs (Figure 3E). Similarly, adhesion to extracellular matrix was increased by 10, 25, 50 and 100 ng/mL GroEL1 treatment (Figure 3F). 

### GroEL1 Increases Senescence That is Mediated by Caspase and MAPK Signaling in EPCs

**Figure 4:**
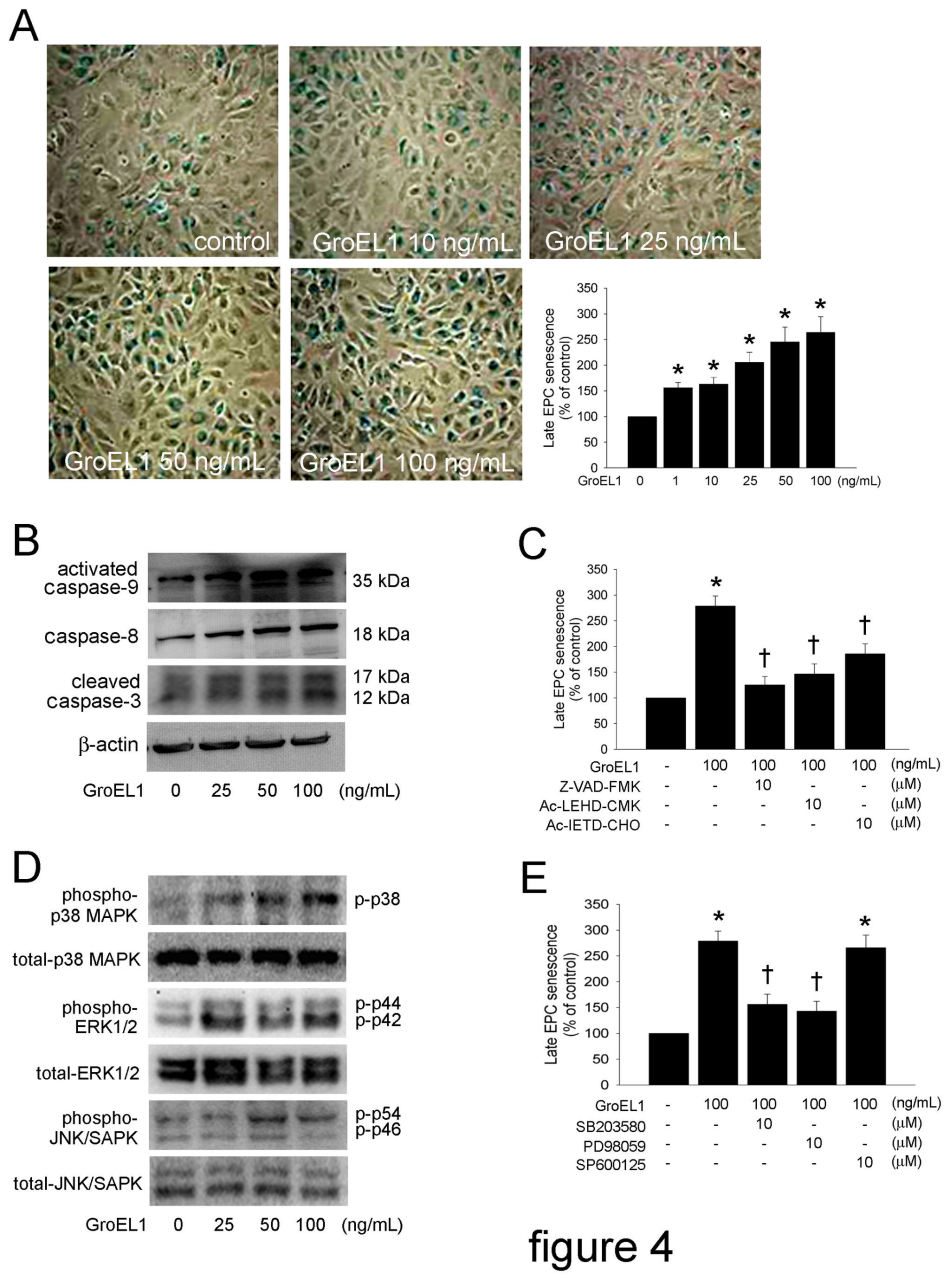
GroEL1 increases senescence, which is mediated by caspases and MAPK signaling in EPCs. (A) Late EPCs were treated with 1-100 ng/mL GroEL1 for 24 hours. Cell senescence was analyzed. The diagram shows the quantification of senescent EPCs. (B) Late EPCs were treated with 25-100 ng/mL GroEL1 for 12 hours. The activation of caspase-9, -8, and -3 were analyzed using western blotting. The β-actin level was used as loading control. (C) Late EPCs were pretreated with 10 μM Z-VAD-FMK, Ac-LEHD-CMK, or Ac-IETD-CHO for 1 hour followed by 100 ng/mL GroEL1 treatment for 24 hours. The cell senescence assay was performed, and the quantified results are shown as a diagram. (D) After treatment of EPCs with 25-100 ng/mL of GroEL1 for 12 hours, the phosphorylation of p38 MAPK, ERK1/2, and JNK/SAPK were analyzed by western blotting. The total p38 MAPK, ERK1/2, and JNK/SAPK levels were used as loading controls. (E) EPCs were pretreated with 10 μM SB203580, PD98059, or SP600125 for 1 hour followed by 100 ng/mL GroEL1 treatment. The cell senescence assay was performed, and the quantified results are shown as a diagram. All data represent the results of three independent experiments and are expressed as the mean±SEM (*p < 0.05 compared with untreated group; †*p* < 0.05 compared with GroEL1-only-treated group).

To evaluate the onset of cellular aging, acidic β-galactosidase was detected as a biochemical marker of acidification, which is typical of EPC senescence [46]. Thus, late EPCs were treated with 10, 25, 50, or 100 ng/mL of GroEL1 for 24 hours. Senescence often started early after 1 ng/mL of GroEL1 treatment (Figure 4A). Exogenous caspases can modulate *ex vivo* formation and senescence of EPCs [47,48]. Therefore, in this study, we investigated whether caspase-9, -8, and -3 regulated senescence in GroEL1-treated EPCs. Western blot analysis showed cleaved caspase-9, caspase-8, and cleaved caspase-3 were increased under GroEL1 treatment in late EPCs (Figure 4B). After 1 hour of pre-incubation with Z-VAD-FMK, Ac-LEHD-CMK, or Ac-IETD-CHO, senescence was significantly decreased in GroEL1-stimulated EPCs (Figure 4C). These findings indicate that EPC senescence may be mediated by caspases. Additionally, senescent EPCs express higher levels of MAPKs [49]. Indeed, western blotting indicated that treatment with GroEL1 altered the activation of p38 MAPK and ERK1/2 (Figure 4D), which effects were inhibited by an inhibitor of p38 MAPK (SB203580) and an inhibitor of ERK1/2 (PD98059). In contrast, GroEL1 did not induce the activation of JNK/SAPK in EPCs; treatment with PD98059, a JNK/SAPK antagonist, also did not prevent senescence of EPCs, suggesting that the JNK/SAPK signaling pathways might not participate in senescence of GroEL1-treated EPCs (Figure 4E).

### GroEL1 Decreases Integrin and E-selectin Expression and Induces Inflammatory Responses in EPCs

**Figure 5:**
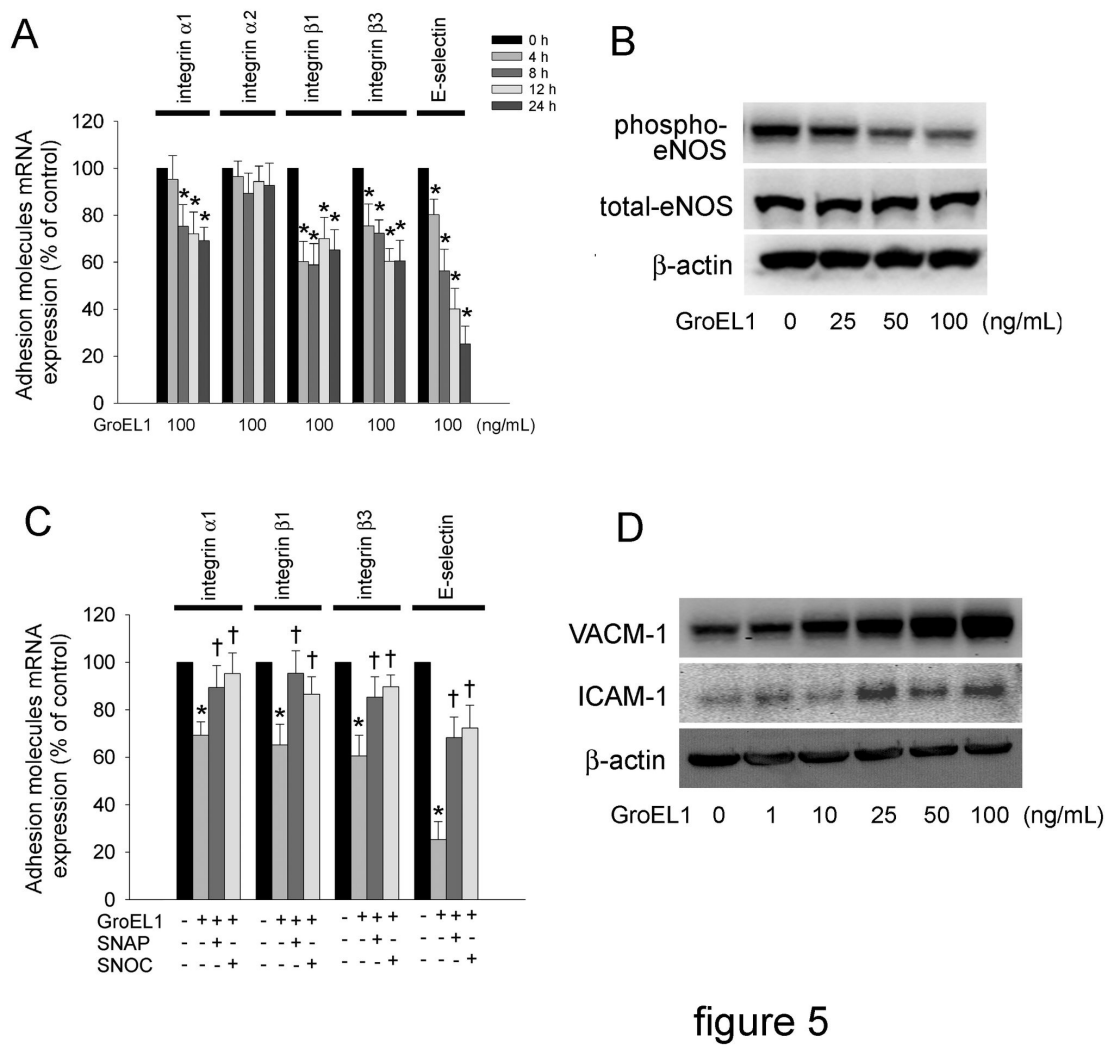
GroEL1 decreases integrin and E-selectin expression and induces adhesion molecule production in EPCs. (A) Late EPCs were treated with GroEL1 (100 ng/mL) for 4-24 hours. Quantitative real-time PCR was also performed for integrin α1, -α2, -β1, -β3, and E-selectin. (B) Late EPCs were treated for 4 hours with 25-100 ng/mL GroEL1. eNOS activation was analyzed by western blotting. The total eNOS and β-actin levels were used as loading controls. (C) EPCs were pretreated with 10 μM SNAP or SNOC for 1 hour followed by 100 ng/mL GroEL1 treatment. Quantitative real-time PCR was performed for integrin α1, -β1, -β3, and E-selectin. All data represent the results of three independent experiments and are expressed as the mean±SEM (*p < 0.05 compared with untreated group, †*p* < 0.05 compared with GroEL1-only-treated group).

*In vitro* and *in vivo* evidence indicates many integrins and selectins function in the regulation of EPC homing and migration during vasculogenesis [50,51]. Integrin α1, -α2, -β1, -β3, and E-selectin are involved in recruitment of EPCs to ischemic muscle [51]. Thus, we investigated whether the expression of integrins was regulated by GroEL1 treatment. Quantitative real-time PCR showed that incubation of cells with 100 ng/mL GroEL1 for 4-24 hours significantly decreased integrin α1, -β1, and -β3 but not integrin β1 mRNA in EPCs (Figure 5A). GroEL1 treatment decreased E-selectin mRNA expression in a time-dependent manner (80.2±6.5% of control after 4 hours, 56.3±9.2% of control after 8 hours, 40.2±8.6 of control after 12 hours and 25.3±7.5% of control after 24 hours). Activation of eNOS plays an important role in EPC function. To determine whether eNOS activation could contribute to the effects of GroEL1 on integrin mRNA expression, cells were pretreated with (±)-S-nitroso-N-acetylpenicillamine (SNAP; an NO donor) or S-nitrosocysteine (SNOC; an NO donor). Western blot analysis showed that incubation with 25, 50, or 100 ng/mL of GroEL1 significantly decreased eNOS phosphorylation at Ser^1177^ in EPCs (Figure 5B). This phenomenon was inhibited by pretreatment with SNAP or SNOC (Figure 5C). VCAM-1 and ICAM-1 have been characterized as proinflammatory mediators that are expressed on EPCs [52]. VCAM-1 and ICAM-1 expression in EPCs were increased by 10, 25, 50, and 100 ng/mL GroEL1 treatment (Figure 5D).

## Discussion

In the present study, we provide evidence that 1) heat shock protein 60 of *C. pneumonia*, GroEL1, impaired the recovery of capillary density, which may be mediated by TLR4 in mice; 2) GroEL1 impaired EPC mobilization and vessel formation as well as eNOS expression in ischemic tissue; 3) GroEL1 administration impaired the migration and vasculogenesis of late EPCs *in vitro*; 4) EPC senescence was enhanced by GroEL1, which was mediated by activation of caspases, p38 MAPK and ERK1/2; and 5) GroEL1 decreased integrin and E-selectin expression but induced inflammatory responses in EPCs. These findings suggest that TLR4 and impaired NO-related mechanisms could contribute to the reduced number and functional activity of EPCs in the presence of *C. pneumonia* GroEL1.

Our study demonstrates that a TLR4-associated mechanism contributes to neovascularization in inflammation-associated angiogenesis in ischemic tissue. Activation of the immune system via TLRs is implicated in angiogenesis, atherosclerosis, and various vascular complications [53]. However, the mechanisms by which immunity and inflammatory responses are involved in angiogenesis of ischemic tissue have not been elucidated completely. Recent studies highlight the critical role of TLRs in the induction of inflammatory responses in ischemic diseases. For example, deficiency of TLR4 protects the myocardium from ischemic injury, whereas modulation of TLR2 induces cardioprotection against ischemic insult. In addition, TLRs are involved in the induction of angiogenesis, modulation of stem cell function, and expression of microRNAs, which are currently important areas in ischemia research [54]. By contract, without exogenous pathogen-associated ligands, TLR4 can be activated by endogenous agonists, such as high-mobility group box 1 (HMGB1), which is produced by damaged tissue or infiltrating immune cells in the injured sites [55]. He et al. used an acute oxygen-induced ischemic retinopathy model in TLR4^−/−^ and WT mice to discover an unrecognized pathway involving HMGB1 and its interaction with TLR4 in angiogenesis. Their results suggest that TLR4 deficiency retards the TLR4-mediated response and downregulates the expression of VEGF, resulting in decreased neovascularization [53]. These results reveal a novel aspect of the multi-faceted TLR biology and may suggest new prospects for using TLR4-related mechanisms to modulate the production of EPCs for clinical use [56]. 

*C. pneumoniae* may play a key role in the development of atherosclerosis [1]. *C. pneumoniae* may induce and accelerate the formation of atherosclerotic lesions in hypercholesterolemic mice or ApoE3-Leiden mice [6]. Our previous study also showed that GroEL1 enhances atherogenesis in hypercholesterolemic rabbits [19]. In our current *in vitro* study, we provide the first evidence that GroEL1 treatment impairs EPC migration and vasculogenesis and enhances EPC senescence by activating caspases, p38 MAPK and ERK1/2. The integrity of the endothelial monolayer plays a pivotal role in atherogenesis. Extensive endothelial cell damage by infection or other cardiovascular risk factors can result in endothelial cell apoptosis with subsequent loss of integrity of the endothelium [30]. It has been suggested that bone marrow-derived circulating EPCs play an important role in endothelial cell regeneration [57]. A reduced level of circulating EPCs independently predicts atherosclerotic disease progression and future cardiovascular events [58], which may support a crucial role for EPCs in modulating the clinical course of various cardiovascular diseases by promoting endogenous vascular repair. The increased prevalence of cardiovascular complications in patients with infection and chronic inflammation may also be attributed to the disturbed balance between increased endothelial injury and hampered endothelial repair processes. 

It is evident that decreased bioavailability of NO produced by eNOS plays a crucial role in the development and progression of atherosclerosis. Our evidence shows that GroEL1 directly inhibited eNOS phosphorylation in EPCs, suggesting that impairment of vascular NO bioavailability by GroEL1 may damage the endothelium. Of note, the expression and phosphorylation of eNOS are essential for the survival, migration, homing, and angiogenesis of EPCs [59]. Our work further indicates that the administration of NO donors can reverse the down-regulation of integrins and E-selectin (essential molecules for EPC homing) in EPCs that are treated by GroEL1, suggesting the critical role of NO in reversing GroEL1-induced dysfunction of EPC homing. Therefore, enhancement of the number and functional capacity of EPCs by increasing NO bioavailability through novel pharmacological strategies could be of potential clinical benefit, especially in subjects with chronic inflammation. MAPKs play a key role in EPC dysfunction [60]. In our study, it was interesting to find that EPCs cultured with GroEL1 showed significantly increased phosphorylation of p38 MAPK and ERK1/2, and administration of p38 MAPK and ERK1/2 inhibitors inhibited GroEL1-enhanced EPC senescence. These findings suggest that GroEL1 might enhance EPC senescence through the down-regulation of eNOS by MAPKs [61]. 

EPCs are thought to release multiple angiogenesis factors [24], which may explain the potent neovascularization after EPC mobilization. However, little is known about the release of proinflammatory factors by EPCs. Zhang et al. showed that paracrine factor release is significantly augmented when EPCs are exposed to the proinflammatory cytokine TNF-α and that TNF-α can induce surface adhesion molecule upregulation in EPCs, as in mature endothelium [52]. Our results show that VCAM-1 and ICAM-1 expression in EPCs were increased by GroEL1 treatment, indicating GroEL1 may induce the inflammatory responses in EPCs that could promote a proinflammatory environment in the vasculature and possibly also damage the endothelium. Recently, Spigoni et al. showed that pioglitazone (an insulin-sensitizing PPARγ agonist) reduces VCAM-1 and ICAM-1 adhesion molecule expression in EPCs, suggesting its potential beneficial cardiovascular effects beyond its anti-hyperglycemic function [20]. These findings suggest the novel pharmacotherapeutic option of modulating EPC paracrine activity in combination with the use of anti-inflammatory agents to treat atherosclerosis. 

Distinguish between endothelial cells and endothelial microparticles were important processes in this study. Endothelial microparticles were shed from the plasma membrane of endothelial cells, which with about 100 nm to 1 μm in diameter [62]. Endothelial microparticles carry endothelial proteins such as vascular endothelial cadherin, PECAM-1, ICAM-1, E-selectin, and integrin. During analysis of flow cytometry, Forware SCatter (FSC) parameter gives a relative size for the cell, and Side SCatter (SSC) parameter is a measurement of the amount of the laser beam that bounces off of particulates inside of the cell. According to the difference size and internal complexity of endothelial cell and endothelial microparticle, we have the opinion about that the FSC and SSC parameters are capable to distinguish them. Although we did not use the DNA staining reagent to identify the endothelial cells, we consider that the affect of endothelial microparticles may be excluded using FSC and SSC parameters.

In conclusion, the *C. pneumonia* heat shock protein 60, GroEL1, impaired the recovery of capillary density, which may have been mediated by TLR4 in mice. Thus, targeting the TLR4 signaling pathway may constitute a novel therapeutic approach to angiogenesis-related diseases. Moreover, GroEL1 enhanced cellular senescence and decreased the functional competence in EPCs. NO-related inflammatory mechanisms could be the main contributor to GroEL1-induced EPC dysfunction. These findings not only give further insight into the complex cellular mechanisms of EPC function in impaired vascular repair and abnormal neovasculogenesis, but they also provide a potential therapeutic target for atherogenesis in *C. pneumoniae* infection. 
